# Hunger, Food Sovereignty and COVID-19 Pandemic: Food Risks During Lockdown

**DOI:** 10.3389/ijph.2023.1605837

**Published:** 2023-11-13

**Authors:** Mónica Juliana Chavarro, Janeth Mosquera-Becerra

**Affiliations:** ^1^ Alliance Bioversity International and CIAT, Cali, Colombia; ^2^ School of Public Health, University of the Valley, Cali, Colombia

**Keywords:** pandemic, lockdown, food, eating behavior, hunger

## Abstract

**Objective:** This article focuses on describing the food scenario of families in Cali (Colombia), where almost half of the city’s population could not guarantee their access to adequate feeding during COVID-19 crisis.

**Methods:** Involved 1. Analyze laws to understand their relationship with access to food in Cali during lockdown; and 2. Identify changes in the eating practices of families from different socioeconomic levels and the strategies used by the city’s public institutions during lockdown.

**Results:** Feeding was not considered from the beginning of the lockdown, which generated a food crisis. Institutional responses were insufficient in quality and coverage, since feeding aid focused on calories and logistic aspects. The solutions implemented by households were guided by collective action and social organization around the community pots.

**Conclusion:** The contrast between food security strategies (focused on availability and access) and food sovereignty (with an emphasis on the collective) shows the need for structural transformations in food policies and in the collective imagination that allow for designing new food models focused on community wellbeing and not on economic growth to future emergencies.

## Introduction

In Colombia, the population remained under different lockdown schemes for 162 days between 22 March and 31 August 2020, due to the COVID-19 pandemic [1]. Food, an aspect of life that had become almost mechanical, became one of the central themes during this period to be addressed, both by families and public institutions. For the former, concerns ranged from “what are we going to eat” to “how do we make it enough for everyone” because their incomes decreased or disappeared; and for the latter, the questions revolved around “how to prevent a crisis during the lockdown.”

**FIGURE 1:**
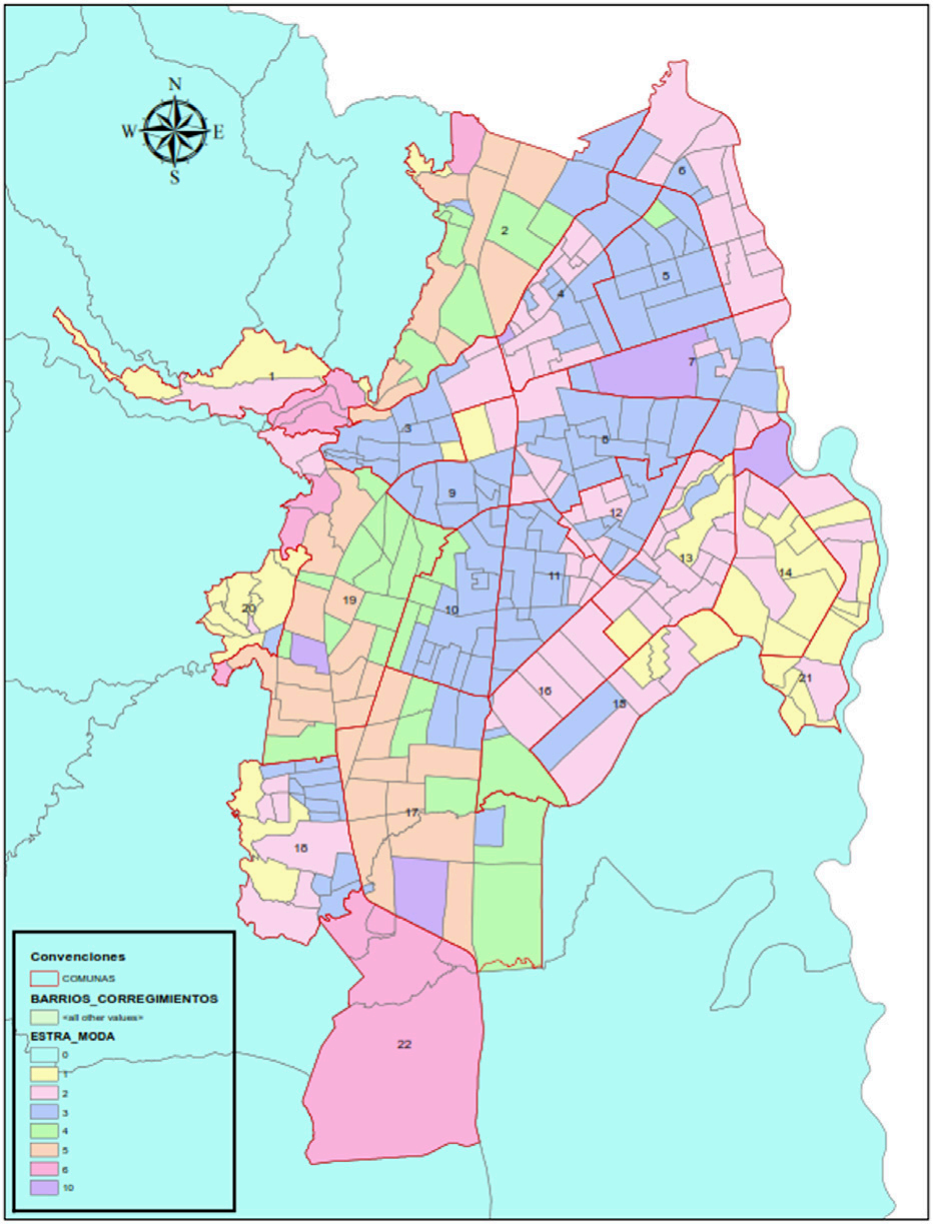
Map of Political division of Santiago de Cali by socioeconomic strata (Food social space in times of pandemic, Cali, Colombia. 2020).

This research was conducted in Cali, the third most densely populated city in Colombia with 2,280,907 inhabitants with a significant Afro-Colombian population [2], located in the southwest of the country, and the main recipient of the migrant population in this area of the country. It also exhibits significant socioeconomic inequality with a GINI index of 0.523, reflected in socio-spatial and racial segregation [3]. Figure 1 is a map of the political division of the city which provides a glimpse of the differences in the spatial distribution according to socioeconomic stratum.

The question that led the research was related to how the food security approach influenced the responses to the food needs of the population of Cali, particularly during the period of lockdown by COVID-19. The answer to this question highlights the need to make a paradigm shift and enable food sovereignty to guide food policies.

This article demonstrated how the government’s strategies to address the demand for food, favored large distribution platforms that market processed and ultra-processed food products, to the detriment of local and grassroots initiatives. To a significant extent, this happened because the local food system has traditionally emphasized in the availability of food (food security), while neglecting other aspects related to food sovereignty such as the origin of the food, the traditions around it, the way of cultivating it, and the social relationships that make it possible [4].

Additionally, local and national food regulations do not provide guidance on how to act during times of crisis. As a contrast, community initiatives emerged that helped counteract the hunger experienced during the lockdown, while also fostering dynamics of solidarity and leadership within communities.

## Methods

To address the research question, a case study was conducted between March and August 2020, months when the lockdown period took place. The study involved: a systematic review of news, policies, and articles focused on the lockdown period; and semi-structured interviews.

**FIGURE 2:**
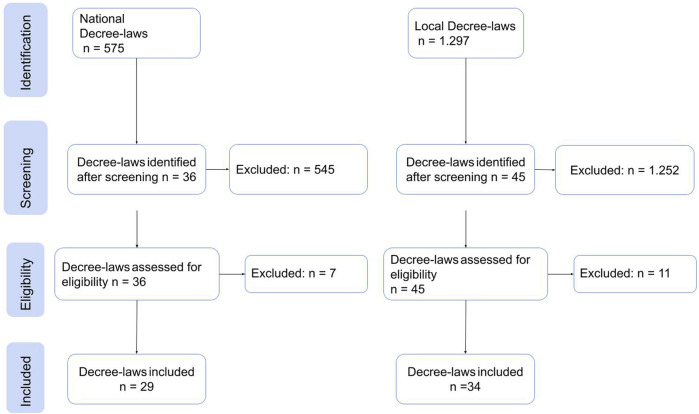
Flowchart Decree-laws selection process (Food social space in times of pandemic, Cali, Colombia. 2020).

**FIGURE 3:**
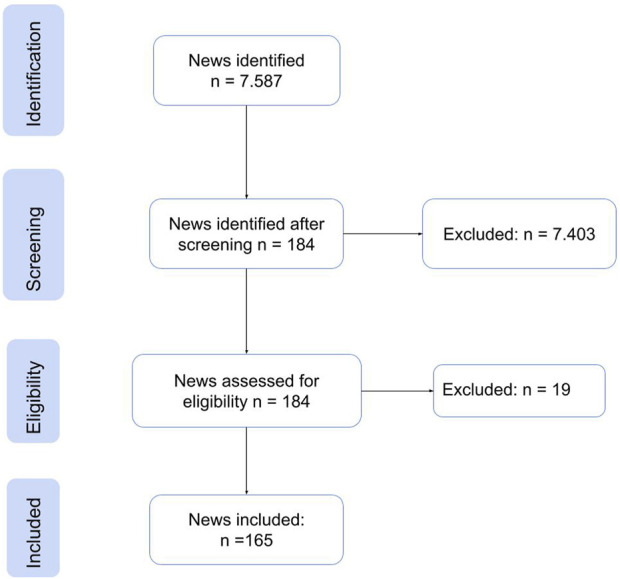
Flowchart News selection process (Food social space in times of pandemic, Cali, Colombia. 2020).

Regarding the selection of policies, emphasis was placed on the Decree-laws issued by the national and local governments and published on their official websites, which were related to food, food supply chain, and the lockdown period. As for the news, a search was conducted in two newspapers, one national and one local, using the keywords “food,” “markets,” and “pandemic,” and those news articles related to the food situation in Cali during the study period were selected. A total of 228 documents were selected from the 9.459 found in the initial search. The selection process followed the scheme proposed by [5] which is summarized in Figures 2, 3 and for both, regulations and news, articles the selection were related to their relevance to food during the lockdown.

The interviews were semi-structured, conducted virtually and had two prioritized groups: on the one hand, families and, on the other hand, food system actors. Families were identified using the snowball sampling technique, prioritizing different family compositions (nuclear, single-parent, extended, single-person) and different socioeconomic strata (considering the impact of the lockdown based on the number of people in the household and their income-generating possibilities). Within the families, the person responsible for food purchases was identified to ask them questions related to changes experienced in food purchase and consumption due to the lockdown. The food system actors, were contacted based on their involvement with food (public officials, NGO members, and retailers). All interviews began with obtaining informed consent, were recorded, and had an average duration of 1 hour. In total, 26 interviews were conducted, 15 with families and 9 with the food system actors.

**FIGURE 4:**
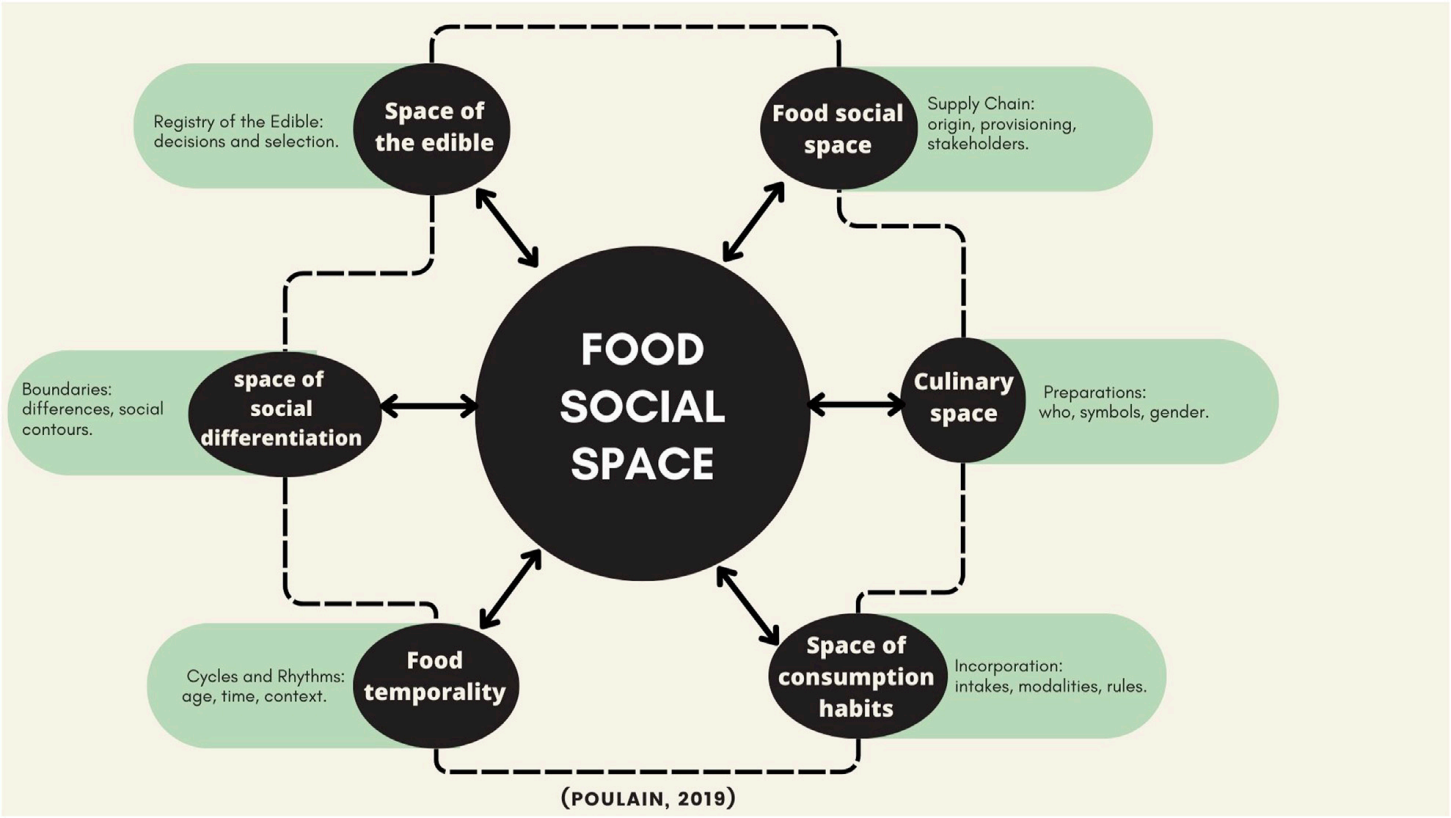
Diagram of the food social space (Poulain, 2019) (Food social space in times of pandemic, Cali, Colombia. 2020).

For the analysis, both interviews (interviews were transcribed) and documents underwent information coding using ATLAS.ti. The codes (constructed following the parameters of [6]) were constructed based on the theory of the Food Social Space [7], (a concept from the socioanthropology of food that places diners and their communities at the center of analysis, emphasizing the interactions between dietary patterns, the social, the psychological, the biological, and the ecological, and integrating the multiple dimensions that give rise to the discourses, imaginaries, and representations intrinsic to the circulation of food, food products, and their meanings) and corresponded to the dimensions it encompasses: space of the edible, food social space, culinary space, space of consumption habits, food temporality, and space of social differentiation, Figure 4 shows a representation of them.

## Results

The analysis of the decree-laws made it possible to identify the strategies proposed by the institutions, which included restrictions on mobility, permits to make purchases and guidelines for food aid provided to families that had lost their income. The news, on the other hand, made it possible to identify the application of the provisions of the decree-laws, as well as the situations that arose, and the strategies that families implemented during this period to guarantee their food. Regarding the interviews, they made it possible to approach the details of what happened within the households: distribution or overload of tasks, food preparation and food acquisition. Tables 1, 2 describe the people interviewed, their socio-demographic characteristics (in the case of families) and their political affiliation (in the case of food system actors).

**TABLE 1:** Characterization of the families interviewed (Food social space in times of pandemic, Cali, Colombia. 2020).

Name	Sex	Age	Commune	Socioeconomic strata	Occupation	Conformation	Persons in the home
Dilia	F	61	21	1	Domestic employee	Unipersonal	1
Jennifer	F	33	1	1	Entrepreneur	Single-parent (mother and child)	2
Diego	M	33	14	2	Economist	Nuclear (father, mother, and child)	3
Juana	F	48	15	2	Domestic employee	Nuclear (father, mother, and child)	4
Lucila	F	72	13	2	Homemaker	Extensive (grandmother, children, grandchildren, great-granddaughter)	6
Landa	F	48	11	3	Secretary	Nuclear (father, mother, and child)	4
Nelly	F	66	7	3	Seller	Nuclear (father, mother, and child)	3
Nelson	M	59	8	3	Architect	Extensive (spouse and wife’s sister)	3
Nubia	F	67	5	3	Homemaker	Extensive (grandmother, children and grandchildren)	6
Oliva	F	54	7	3	Homemaker	Nuclear with a visiting mother-in-law	3
Esperanza	F	70	17	4	Retired	Unipersonal	1
Leonilde	F	67	2	4	Homemaker	Single-parent (mother and child)	2
Constanza	F	50	17	4	Manager	Nuclear (father, mother, and child)	4
Alejandro	M	40	19	6	Lessor	Unipersonal	1
Laura	F	65	22	6	Occupational therapist	Nuclear with son and daughter-in-law	4

**TABLE 2:** Characterization of Food Social Space actors interviewed (Food social space in times of pandemic, Cali, Colombia, 2020).

Id dentification	Sex	Organization	Job title
A1	M	Secretariat of Economic Development	Ex Secretary of Economic Development
S	F	Secretariat of Economic Development	Food Safety Team Leader
R	M	Social Welfare Secretariat	Community Kitchens program coordinator
A2	F	Food Bank	Coordinator
F	F	Social Welfare Secretariat	Ex Secretary of Social Welfare
F1y	M	FENALCO	Telephone sales platform supervisor
L	F	Market place	Salesperson
A3s	M	Market place	Salesperson
F2	F	Market place	Salesperson

With the analysis carried out, a narrative was elaborated using direct quotations from interviews, news, and decree-laws, looking to, a) show how food was guaranteed to vulnerable people before confinement in Cali and, b) show the dynamics, situations and strategies that were experienced and implemented by institutions and families during the confinement period. Two sections are presented below, one for each aspect mentioned.

### Cali’s Food Security Strategy Pre-Pandemic

Before the pandemic, there was already a situation of food insecurity in the city, which was addressed through developing different programs: i) the school feeding program (PAE by its Spanish initials), for children and adolescents attending educational institutions; ii) the community kitchens, located in strategic areas of the city to cover the most vulnerable population (in conditions of poverty and extreme poverty), and iii) efforts made by the social service providers and food banks, whose main beneficiaries are legally-constituted social organizations, community kitchens, foster homes, kindergartens and educational institutions [A2. Personal interview. February 22, 2021].

The PAE is the oldest of these programs, having its origins in 1941 [8] and operated through the delivery of snacks and lunches to students in public institutions, seeking to guarantee a daily minimum individual provision of calories and with the certainty, that the food the beneficiaries eat there may be the only food they receive during the day. The community kitchens, originated in 1990, when institutional support began to be given to community pots, (initiatives where food is prepared in large quantities and are led by people who organize themselves in solidarity to prevent their own or others’ hunger) created by civil society, which later became the first community kitchens, and their operation was guaranteed through agreements with established companies with the economic capacity to accept the difference in payment times and who also have the capacity to deliver everything on the food menu. “I have a supplier who is able to organize everything in a single market and in a single trip I get everything at the canteen” [R. Personal interview. February 18, 2021].

Finally, the food bank formally began its operation in 2000 and its operating structure is based on receiving donations of either money or food that for some reason does not meet market quality standards (presentation, labelling) or that due to its high state of maturity must be used before it is lost and delivered to the beneficiary population. As for the cash donations, they are used to buy food in large distribution platforms “with the cash donations, what we do is automatically buy, because we have suppliers that give us very good prices, so we buy, bring, pack, balance the markets and go out to deliver” [A2. Personal interview. February 22, 2021].

Considering that both the community kitchens and the PAE are among the beneficiaries of the Food Bank and that a large amount of the Bank’s food comes from the large platforms, we can affirm that Cali’s food strategy before the pandemic was highly dependent on the large distribution platforms for its functioning. This situation, far from changing during the pandemic, increased during the period of lockdown.

### Cali’s Food Strategy During Lockdown

The city’s food strategy began with the prioritization of street dwellers and public nursing homes because the confinement was initially planned as a 3 days pilot [9]. Later, with the national declaration of the confinement period for a period of almost 20 days, the approach was modified to respond to a vulnerable population prioritized according to socio-demographic variables (socioeconomic stratum, spatial location, age group and informal jobs).

The mayor’s office designed a strategy that consisted of delivering food aid to families that could not guarantee access to food; this aid was built on the basis of the basic minimum calorie requirement for an adult, with a caloric intake of 1959.62 kilocalories, which was enough for 4 days for a household of four people and did not include all food groups, as it did not include neither fruits nor vegetables in its composition [A1. Personal interview. February 15, 2021].

The implementation of the strategy was based on the previously described experience of the mayor’s office in food procurement, according to which the almost exclusive source of supply was the large distribution platforms: customers with an infrastructure large enough to offer the food demanded and with the financial capacity to wait for months for payments, but who did not have the supply of perishable food, thus reaffirming the decision to maintain food aid without these food packages.

However, there were other actors within the city’s food system that could have provided fruit and vegetables to the food aids, such as the central supply center, marketplaces, and producers in the surrounding rural area. Each of these was ruled out for logistical, legal, or economic reasons, respectively. In the first case, the central supply center required greater logistics for the mayor’s office [A1. Personal interview. February 15, 2021]; in the second, the marketplaces had legal entities that were not designed to contract with the State such as, foundations that are not authorized to receive funds [S. Personal interview. February 19, 2021]; and in the third, the producers did not have the economic capacity to defer payments for months at a time.

Once the delivery of food aid and its composition had been defined, the idea was to expand coverage through complements: the PAE, unable to deliver prepared food in institutions, also delivered food rations in educational institutions [10]; the operation of community kitchens was enabled so that one person per family could claim a daily ration for their family unit; and the food bank adapted its model to begin to respond to organized civil society groups that were formed as the hunger situation worsened.

The worsening of the situation occurred for two main reasons: a) the underestimation of the population impacted by the lockdown, since the considered variables excluded those many people with formal jobs who could no longer work and who therefore lost the capacity to generate income to access food on their own “of course, all the containment measures forced many people to lose their jobs or their income and need support or food assistance from the government” [R. Personal interview. February 18, 2021]. And b) the high demand experienced by the large distribution platforms, exercised by families who made panic purchases [11] and by institutions seeking to deliver food aid to the vulnerable population. Faced with this situation, the response of the large platforms was to prioritize their usual clients (families), leaving the institutions with many commitments and no capacity to respond, both for market deliveries and for delivery of rations to community pots [A1. Personal interview. February 15, 2021].

The lack of compliance triggered different community responses as the lockdown progressed. The first of these was the use of red rags, by means of which families signaled the hunger they were experiencing, without leaving their homes, with the idea of receiving help from the authorities or neighbors. “There is not a house here that does not have a red rag in its window, a sign of help to be able to feed themselves... we are hungry, and we need help” [12].

“*We have a very serious problem because the mayor's office is not able to deliver to all the people who were at that time taking out red rags, and you could see red rags all over the city in April and May*” [A1. Personal interview. February 15, 2021]

Faced with the mayor’s office’s inability to respond to this first call, many people took to the streets, some to demonstrate and protest [13] and others to tour the city, especially in the residential units of the higher socio-economic strata “... they do it in the doorways of the units… with megaphones and then they say: ‘look, I live in such, and such a place and we don't have enough to eat… the situation is difficult, help us with money, with food, with whatever” [Constanza. Personal interview. August 29, 2020], and many others began to organize around community pots.

Of these strategies, the ones that most served the families were community pots, as they were platforms for social interaction and articulation [14]. Community pots were identified by the institutions as an opportunity to deliver food with far fewer logistical demands, while allowing the delivery of a wider range of food to a larger number of people.

[...] The community pots allowed us to deliver, not food aids, but rice, potatoes [...] eggs, chicken and people also put in their work…... and meals were made [A1. Personal interview. February 15, 2021].

The mayor’s office delivered food aids to the community pots and identified points in the city where new community pots could be established and had them functioning for 2 months [R. Personal interview. February 18, 2021]. Additionally, they complemented their food strategy with the delivery of exchangeable vouchers in large distribution platforms and in some neighborhood shops, with which families could decide what to buy as long as it was food or cleaning supplies [15].

Finally, both national and local institutions focused on extending exceptions so that people could gradually resume their work and once again secure access to food on their own. This strategy was identified as “economic reactivation” and eased the burden:

“We served about 100 community pots, and we were serving them weekly, until they themselves were deactivated, because they emerged and multiplied at the time of the closure, but when the opening started it started to go down. And they remain because there have always been community pots and we have always attended to them, but the usual ones remain” [A2. Personal interview. February 22, 2021].

In summary, the 162 days of lockdown brought the city, its families, and its institutions to a standstill. No one was adequately prepared for the challenges that arose, and there were no suitable food policies in place to address the situations experienced. Moreover, the decisions that were taken failed to respond to the reality unleashed by the lockdown and led to constantly changing strategies in search of the most suitable one, which, considering scope and quality, ended up being the one focused on the community kitchens.

## Discussion

The food strategy built in Cali during the lockdown period by the institutions, from a food security approach, was insufficient to solve the pandemic problems, as it did not respond effectively to the food needs of the population. This can be explained by the economic orientation that prioritized large distribution platforms, the lack of flexibility regarding possible alternatives that would allow for variety and quantity in the aid delivered, and the lack of knowledge of the heterogeneity of the population living in the city.

Prior to the lockdown, the city had a food context in which the large distribution platforms were the main suppliers of food, which led to the prioritization of processed and ultra-processed food products, to the detriment of other food groups, and long supply/value chains to the detriment of shorter ones. This phenomenon increased during the period of lockdown with the delivery of food aid that was insufficient in coverage and quality, as it did not reach all the households in need and did not have adequate nutritional intake.

Regarding coverage, priority was given to the individual delivery of aid, which is explained by the characteristics of the COVID-19 contingency, during which the aim was to prevent contagion by means of social distancing. However, as it did not manage to cover the entire population, nor guarantee the frequency of delivery, collective responses such as marches and protests were triggered, which contravened social distancing rules.

The second problem had to do with food supply, since the aid was structured prioritizing non-perishable foods, thus depriving families of the possibility of consuming fruit, vegetables, and dairy products. The argument for this food scheme was that there were no logistical, legal, or economic options for contracting other suppliers. This argument showed a lack of flexibility in the regulations to respond to vital issues such as food and was manifested in the limitation of not being able to buy perishable foods from the marketplaces that would have more effectively responded to the nutritional and cultural characteristics of the consumers.

However, successful experiences in other countries show that it was possible to buy directly from small producer families, ensuring economic stability in rural areas and healthy food supply for urban families. This is the case in Brazil and Costa Rica, where, despite facing the same legal, logistical and financial challenges [16, 17], they managed to implement a strategy that consisted of purchasing food produced by family farming to supply school feeding programs, which promoted the economic dynamism of producer families and guaranteed the supply of fresh and nutritious food for vulnerable families [18–20].

These examples show that alternatives did exist when designing strategies and that what was required was a stronger political will to make the necessary adjustments to guarantee supplies of quality food for urban families and adequate income for rural families. They also highlight the lack of tools in the public policies that guide food and their implementation mechanisms (PSAN, PNSAN, OSAN, CISAN), which do not have guiding components for times of crisis and did not participate in the construction of food strategies at the national or local level.

This lack of functional public policies is reinforced by the high degree of ignorance of the vulnerability of the city’s households, since the population and its food conditions are not clear to the institutions, which meant that the vulnerable population expanded over time, transcending economic and spatial variables.

The heterogeneity of the population is an extremely important factor, not only in atypical contexts, but above all in the design of food policies and programs, in such a way that they allow for the inclusion of cultural characteristics from food production to consumption.

On the other hand, the dependence on a single supplier and the pressure that both the aids and the markets carried out by families exerted on the supply of large platforms generated a shortage that made it impossible to continue with the delivery of food aid, triggering a series of community reactions that culminated in the community pots.

These community demonstrations, built from a solidarity and collective approach, achieved in a very short time what the institutional system had failed to achieve in months: guaranteeing at least a daily ration of nutritious and varied food for many families who not only benefited, but also participated actively in their management and existence.

It is precisely this new approach that the community cooking pots bring to the social panorama, which is part of any proposal for food sovereignty, for which the construction of networks, the prioritization of aspects beyond economic ones and the recognition of the heterogeneity of the population, are necessary pillars to be considered to achieve fair food that prioritizes life over profitability.

The contrast between food security strategies (focused on availability and access) and food sovereignty (with an emphasis on the collective) shows the need for structural transformations in food policies and in the collective imagination that allow for designing and implementing new food models focused on community wellbeing and not on economic growth.
